# Emerging infectious diseases--Brazil.

**DOI:** 10.3201/eid0401.980101

**Published:** 1998

**Authors:** H Momen


### International Editors update

In this new section, we welcome commentary and updates from our newly formed Board of International Editors. (See inside front cover for a list of names.)

### Emerging Infectious Diseases—Brazil

**Hooman Momen**
Instituto Oswaldo Cruz, FIOCRUZ, Rio de Janeiro, Brazil

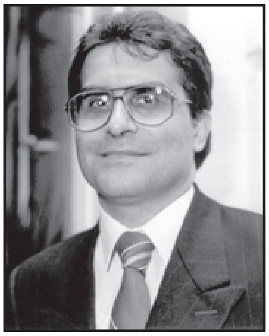

Dr. Momen is a senior research scientist; head of the Department of Biochemistry and Molecular Biology, Oswaldo Cruz Institute, FIOCRUZ; and Editor of Memorias do Instituto Oswaldo Cruz. His research interests include biochemical systematics of pathogenic microorganisms. Dr. Momen is temporary adviser to both WHO and PAHO at several technical consultations and scientific working groups.

Brazil's large size (more than 8.5 million km$^2$ and 150 million population) and inadequate public health infrastructure pose a considerable challenge in assessing the status of emerging infectious diseases. Under the current system, most infectious diseases are not notifiable. In diseases for which notification is required, underreporting is common and varies widely by region and disease, and notification is often delayed, which causes the data to be revised frequently. Moreover, in hospital and clinical settings, the etiologic agent of an infectious disease is often not identified. For example, more than a million hospital admissions are recorded per year by the public health system under parasitic and infectious diseases (the category excludes AIDS and respiratory illnesses); of these diseases, more than 70% are diagnosed as ill-defined intestinal infections and a further 10% as food poisoning and septicemia, both without identification of the etiologic agents. For these reasons, the numbers reported here (1996 data, unless otherwise stated) may not reflect the true numbers of cases, and only diseases whose prevalence has changed markedly in recent years will be included in the review.

Address for correspondence: Hooman Momen, Instituto Oswaldo Cruz, FIOCRUZ, Av. Brazil 4365, Rio de Janeiro 21045-900, Brazil; fax: 55-21-590-3545; e-mail: hmomen@gene.dbbm.fiocruz.br.

### Parasitic Diseases

### Malaria

Among parasitic diseases, malaria causes the most illness, approximately half a million cases annually, nearly all from the northern (Amazon) region. The disease has been controlled in the remainder of the country for years. Where systematic and sustained efforts have been made in the Amazon, control has also been successful. Due to the different epidemiologic situations in the Amazon, no single strategy is effective. Worrying developments in this region include the establishment of the disease in the periphery of the principal cities and the increase in drug-resistant parasites.

### American Trypanosomiasis

American trypanosomiasis (Chagas disease) is the most lethal parasitic disease in Brazil, causing more than 5,000 deaths per year. Several million people are chronically infected carriers of this disease, for which there is still no effective treatment. A very effective control program against the principal insect vector, *Triatoma infestans*, and improved control of the blood supply have reduced the incidence of new cases to a very low level. Factors that continue to make the disease a re-emerging threat include secondary vectors, forest clearance, and congenital transmission.

### Leishmaniasis

Leishmaniasis (cutaneous and visceral) has increased in incidence in recent years. This increase is modified by apparent cyclic variations of undetermined cause (cyclic duration for the visceral form has been estimated at 10 years). The disease has increased not only in the usual areas of forest and recent clearance, but also in areas of traditional colonization as well as in the urban areas of the northeast. One factor involved in the spread of this disease, uncontrolled urban growth due principally to rural migration, has led to pockets (normally on the periphery of the cities) of extreme poverty that favor the spread of many diseases.

### Viral Diseases

### Dengue

Among the notifiable viral diseases, dengue causes the most illness. Its mosquito vector, *Aedes aegypti*, was reintroduced into Brazil in the 1970s; large outbreaks followed in the next decade. Since the vector's reintroduction, more than 700,000 cases have been reported (180,000 cases in 1996). The presence of dengue-1 and -2 indicates that cases of hemorrhagic dengue are also occurring. *Ae. aegypti* is now present in all states, and a plan has been formulated by the federal ministry of health to eradicate the vector, although plan implementation has met with operational difficulties. The widespread distribution of *Ae. aegypti*, also the vector of yellow fever, in areas where this disease was considered endemic poses a serious risk for the reemergence of the epidemic form of yellow fever. Vaccination and sanitation campaigns earlier in the century considerably reduced the incidence of the disease (fewer than 100 cases per year—the last urban cases were reported in 1942).

### Measles

Measles—a viral disease that was controlled and nearly eradicated—reemerged with a vengeance in 1997. The outbreak began in the state of São Paulo, where only 15 cases of measles were confirmed in 1996, and has spread to other states. As of October 1997, 61,000 cases (48,000 in São Paulo) have been reported, and 17,000 (13,000 in São Paulo) have been confirmed. Most cases have occurred in young adults under 30 years of age. The vaccination campaign has been reformulated in response to the outbreak, and a national campaign has been launched. Given Brazil's history of success in organizing such campaigns, it is likely that the outbreak will be rapidly contained and that the effort for measles eradication will be resumed.

### Other Viral Diseases

Other viral diseases include AIDS, with more than 17,000 new cases registered in the last year. The number of deaths due to AIDS was 30% lower in the first half of 1997 than in the first half of 1996. This decrease is attributed to the introduction of new antiviral drug combinations. The spread of the AIDS epidemic, particularly into rural areas, has resulted in coinfections with different infectious agents, producing a variety of novel pathologies. Several subtypes of HIV, as well as novel recombinations, have been reported.

Enteric transmission of hepatitis continues; hepatitis A and D comprise most of the cases, although the peak incidence is now in young adults rather than in young children. The incidence of hepatitis B is declining as a result of vaccination, while that of hepatitis C is increasing. A high prevalence of hepatitis D exists in some regions of the Amazon, where in conjunction with hepatitis B, it is believed to be the principal cause of Labrea black fever.

Influenza is a major concern. Imported vaccine is expensive and not generally available, and the proportion of elderly people (and the threat for a large-scale epidemic) is increasing.

The vast forests that still cover large areas of Brazil are home to many known and unknown viruses. The Evandro Chagas Institute in Belem alone has isolated more than 183 new arboviruses. The increasing exploitation of sylvatic resources put humans in direct contact with these viruses, but because of inadequate or nonexistent medical facilities (entire municipalities still lack a single medical doctor), fevers and even deaths often are not diagnosed or are misattributed. Among new viruses causing fatal infections recently discovered in Brazil are Rocio virus, which causes encephalitis, and Sabiá virus, which causes hemorrhagic fever. Several cases of hantavirus infection have also been reported, but on these and other occasions, the lack of a biosafety level 4 laboratory in the country impeded further work.

### Bacterial Diseases

Enteric bacterial infections are an important cause of illness in Brazil. National figures on prevalence are not available, except for cholera. However, an alarming increase has been reported in antibiotic-resistant strains.

### Cholera

The cholera epidemic in Brazil started with the reemergence of the disease in Latin America in 1991, reached a peak of more than 60,000 confirmed cases and 670 deaths in 1993, and then declined to 1,000 cases in 1996. In 1997, the disease resurged with more than 5,000 reported cases and 2,600 confirmed cases. The reawakening of epidemiologic research in cholera caused by its resurgence led to the discovery of a new biotype of *Vibrio cholerae* in the Amazon region. This biotype is of the O1 serotype; it has distinct multilocus enzyme electrophoresis and RAPD profiles from other pathogenic O1 *V. cholerae*. About 50 isolates have been made from cases of diarrhea in the upper Amazon (Solimoes) River. The microbe apparently lacks the principal known virulence factors (e.g., the toxin gene cassette and the major colonization factor, TCP); however, some isolates present a cytotoxic effect for Y-1 cells.

### Mycotic Diseases

Fungal infections, including histoplasmosis, paracoccidioidomycosis, and cryptococcosis, occur; however, their cause is often unknown and even if a diagnosis is made, the diseases are not reported.

### Public Health Infrastructure

The emerging infectious disease picture in Brazil will not change markedly without a sustained and determined effort to improve the country's public health infrastructure. The existing, generally passive epidemiologic surveillance system produces information that arrives too late to be effective; however, a number of measures, if implemented immediately, can mitigate the impact of any future epidemic: a containment laboratory (biosafety level 4) that can handle known and unknown microbes of high virulence; at least one infirmary, properly designed and fully equipped, to treat highly contagious and virulent diseases. The current lack of this facility poses a great danger to the population should an outbreak of such a disease occur.

The financial, technical, and human resources needed to activate these measures already exist. In infectious diseases, Brazil has a long tradition of fruitful international collaboration that can be tapped for additional support. The success of national vaccination and sanitation campaigns in the eradication and control of some infectious diseases at the beginning of this century and in more recent times demonstrates that much can be accomplished. Brazil can become a successful model for other developing countries.

### Acknowledgments

I thank the Fundação Nacional de Saude for providing data on different diseases; Drs. H. Schatzmayr, J.R. Coura, and P. Mason for interesting discussions and criticisms of the manuscript; and Drs. Ana Gaspar, Clara Yoshida, Ana Carolina Vicente, and Marilda Siqueira for useful information.

