# Correction: Vol. 4, No. 1

**DOI:** 10.3201/eid0402.C10402

**Published:** 1998


limitations in our knowledge. Additional basic and operational research was strongly urged. Some participants expressed concern over the use of the term "elimination," on the grounds that the distinctions between elimination and control and elimination and eradication were unclear. Further discussion and possible revision of these terms were recommended.

In summary, the conference provided a multidisciplinary forum for addressing issues around elimination and eradication and their relationship to sustainable health development. There was widespread agreement that an eradication program could have many positive effects on systems development and that explicit efforts should be made to maximize these positive effects as well as to minimize any negative effects. Community mobilization and organization should be seen as a component of sustainable health development, with the additional potential for disease control and eradication. The conclusions and recommendations of the conference should be brought to other forums to expand international health goals and strengthen the mutual ties between sustainable health development and disease control, elimination, and eradication.

For further information, contact Dr. Walter R. Dowdle, 404-371-0466, e-mail: wdowdle@taskforce.org; or Dr. Richard Goodman, 404-639-7400, e-mail: rag4@cdc.gov.

**Dick Conlon**
Task Force for Child Survival
Carter Center, Atlanta, Georgia, USA

Conference sponsors included Burroughs Wellcome Fund; CARE; The Carter Center; Centers for Disease Control and Prevention (CDC); CDC Foundation; Children's Vaccine Initiative; The Edna McConnell Clark Foundation; The Fogarty International Center; Glaxo Wellcome; International Life Sciences Institute (ILSI); International Union of Microbiological Societies (IUMS); Merck & Co., Inc. Vaccine Division; National Countil for International Health (NCIH); National Institute of Allergy and Infectious Diseases (NIAID); Pan American Health Organization (PAHO); Pasteur Merieux Connaught USA; The Rockefeller Foundation; Rollins School of Public Health of Emory University; The Task Force for Child Survival and Development; United National Children's Fund; United National Development Programme (UNDP); The World Bank; World Federation of PUblic Health Associations (WFPHA); World Health Organization; and Wyeth-Lederle Vaccines and Pediatrics.

### New and Reemerging Infectious Diseases: A Clinical Course

### Atlanta, Georgia, USA, June 13-15, 1998

Jointly sponsored by the Emory University School of Medicine, Centers for Disease Control and Prevention, and National Foundation for Infectious Diseases, New and Reemerging Infectious Diseases: A Clinical Course focuses on the epidemiology, recognition, treatment, and management of new and reemerging infectious diseases. The course will bring together the foremost infectious disease clinicians and epidemiologists to present pertinent information on emerging infections and prospective therapeutic agents.

For more information, contact Kip Kantelo at NFID, 4733 Bethesda Avenue, Suite 750, Bethesda, MD 20814-5228, USA, tel: 301-656-0003, fax: 301-907-0878, http://www.nfid.org/nfid, e-mail: kkantelo@aol.com.

### Erratum

### Vol. 4, No. 1

In the article, "Emerging Infectious Diseases—Brazil," by Hooman Momen, on page 3 the last sentence of the next-to-the-last paragraph should read, "The existing, generally passive epidemiologic surveillance system produces information that arrives too late to be effective; however, a number of measures, if implemented immediately, can mitigate the impact of any future epidemic: a containment laboratory (biosafety level 4) that can handle known and unknown microbes of high virulence; at least one infirmary, properly designed and fully equipped, to treat highly contagious and virulent diseases (the current lack of this facility poses a great danger to the population should an outbreak of such a disease occur); and a mobile multidisciplinary task force, including epidemiologists, microbiologists, entomologists, and clinicians, ready to investigate suspected disease outbreaks on short notice."

In the article, "International Editors Update: Emerging Infectious Diseases—Brazil," by Hooman Momen, on page 3 the last sentence of the next-to-the-last paragraph should read, "The existing, generally passive epidemiologic surveillance system produces information that arrives too late to be effective; however, a number of measures, if implemented immediately, can mitigate the impact of any future epidemic: a containment laboratory (biosafety level 4) that can handle known and unknown microbes of high virulence; at least one infirmary, properly designed and fully equipped, to treat highly contagious and virulent diseases (the current lack of this facility poses a great danger to the population should an outbreak of such a disease occur); and a mobile multidisciplinary task force, including epidemiologists, microbiologists, entomologists, and clinicians, ready to investigate suspected disease outbreaks on short notice."

